# Asymptomatic Ileal Neuroendocrine “Carcinoid” Tumor Incidentally Diagnosed on Colorectal Cancer Screening Colonoscopy: Does Routine TI Intubation Matter?

**DOI:** 10.1155/2021/6620036

**Published:** 2021-02-03

**Authors:** Ali Zakaria, Lynna Alnimer, Gregory Byrd, Marc Piper, Michael Raphael, Bradley Warren, Michael Piper

**Affiliations:** ^1^Division of Gastroenterology, Ascension Providence Hospital, Michigan State University/College of Human Medicine, Southfield, Michigan, USA; ^2^Department of Internal Medicine, Ascension Providence Hospital, Michigan State University/College of Human Medicine, Southfield, Michigan, USA; ^3^Department of Internal Medicine, Ascension Macomb-Oakland Hospital, Warren, Michigan, USA

## Abstract

Gastrointestinal neuroendocrine tumors (GINETs) (also known as “carcinoids”) are rare tumors with reported incidence of up to 6.98 per 100,000 which has increased significantly due to the increased detection on imaging and endoscopy. They are most commonly located in the small bowel, particularly the terminal ileum. Patients with small bowel NETs may present with abdominal pain, diarrhea, or carcinoid syndrome. However, the disease is mostly asymptomatic, and patients are usually diagnosed incidentally during routine colonoscopy. Although the ileum is the most common site for GINETs, terminal ileal (TI) intubation is not always completed during routine colonoscopy. With terminal ileum intubation being successful in at least 70% of colonoscopies and the rate of neuroendocrine tumor detection 0.1–1% of those intubations, one critical question remains unanswered: should terminal ileal intubation be considered a part of the definition of a complete colonoscopy? Herein, we present nine cases of NETs found incidentally on routine colon cancer screening colonoscopy in asymptomatic patients. This case series adds to the sparse literature and highlights the importance of TI intubation technique in early detection of small bowel NETs which could potentially affect the outcome.

## 1. Introduction

Gastrointestinal neuroendocrine tumors (GINETs) (also known as “carcinoids”) are rare tumors. The incidence has increased significantly due to the increased detection on imaging and endoscopy. They are most commonly located in the small bowel, particularly the terminal ileum. The disease is mostly asymptomatic, and patients are usually diagnosed incidentally during routine colonoscopy. Although the ileum is the most common site for GINETs, terminal ileal (TI) intubation is not always completed during routine colonoscopy. Herein, we present nine cases of NETs found incidentally on routine average risk screening colonoscopy to highlight the importance of TI intubation technique in early detection of small bowel NETs which could potentially affect the outcome.

## 2. Cases

**Case 1 .**

A 66-year-old male patient underwent surveillance colonoscopy for CRC due to personal history of colon polyps. A 20 mm submucosal lesion was found on terminal ileal intubation with histopathology revealing carcinoid tumor. His initial lab workup revealed a 5-hydroxyindoleacetic acid (5-HIAA) level of 3 mg\gCR and a chromogranin A of 7 ng\ml. A CT scan abdomen\pelvis was performed and showed a non-obstructing mass in the terminal ileum, with no evidence of metastasis. He underwent mini-laparotomy with right hemicolectomy, and histopathology confirmed the diagnosis of well-differentiated neuroendocrine tumor infiltrating muscularis propria with metastatic tumor in 4 out of 10 resected lymph nodes (T2N1M0). A follow-up lab workup revealed 5-HIAA level of 3 mg\gCR and chromogranin A of 40 ng\ml. He had repeated colonoscopy after 2 years with normal neo-ileum.

**Case 2 .**

A 57-year-old female patient underwent average risk screening colonoscopy. An 11 mm submucosal lesion was found on terminal ileal intubation with histopathology revealing carcinoid tumor. Her initial lab workup revealed a 5-HIAA level of 4 mg\gCR and a chromogranin A of 101 ng\ml. A CT scan abdomen\pelvis was performed and showed a mesenteric nodule measuring 9 mm adjacent to the terminal ileum, with no evidence of metastasis. She underwent laparoscopic right hemicolectomy, and histopathology confirmed the diagnosis of low-grade neuroendocrine tumor involving the muscularis propria with metastatic tumor in 7 out of 19 resected lymph nodes (T2N1M0). A follow-up lab workup revealed 5-HIAA level of 2.4 mg\gCR and chromogranin A of 99 ng\ml. She had repeated colonoscopy after 1 and 3 years with normal neo-ileum.

**Case 3 .**

A 57-year-old male patient underwent average risk screening colonoscopy. A 15 mm lesion was found on terminal ileal intubation with histopathology revealing carcinoid tumor. His initial lab workup 5-HIAA and chromogranin were unavailable. A CT scan chest\abdomen\pelvis was performed and showed a faint enhancement of the terminal ileum with no evidence of metastasis. He underwent a laparoscopic ileocolectomy, and histopathology confirmed the diagnosis of malignant carcinoid tumor with metastasis to 2 out of 15 resected lymph nodes (T4N1M0). A follow-up lab workup revealed a 5-HIAA of 2 mg/gCR and chromogranin A of 62 ng/ml. He had repeated colonoscopy after 1 and 4 years with normal neo-ileum.

**Case 4 .**

A 69-year-old female patient underwent average risk screening colonoscopy. A 20 mm lesion was found on terminal ileal intubation with histopathology revealing carcinoid tumor. Her initial lab workup regarding 5-HIAA was unavailable and the chromogranin A was 48 ng/ml. A CT scan abdomen\pelvis was performed and showed 10 mm lobulated excrescence involving terminal ileum and a nonspecific 1.8 × 0.9 cm central mesenteric lymph node. She underwent laparoscopic right hemicolectomy, and histopathology confirmed the diagnosis of well-differentiated NET with metastasis to 3 out of 27 resected lymph nodes (T1N1M0). A follow-up lab workup revealed a 5-HIAA and chromogranin A of 3 mg/gCR and 70 ng/ml, respectively. She had repeated colonoscopy after 2 years with normal neo-ileum.

**Case 5 .**

A 55-year-old female underwent surveillance colonoscopy for CRC due to personal history of colon polyps. A 10 mm polyp was found on terminal ileal intubation with histopathology revealing carcinoid tumor. Her initial lab workup revealed a 5-HIAA of 3 mg/gCR and chromogranin A of 1152 ng/ml. A CT scan chest\abdomen\pelvis and NM octreotide (tumor localization) scan were performed and showed no definitive lesions suspicious for metastasis. She underwent a right laparoscopic hemicolectomy, and histopathology confirmed the diagnosis of carcinoid invading the submucosa with metastasis to 6 out of 29 resected lymph nodes (T1N1M0). A follow-up lab workup revealed a chromogranin A of 1160 ng/ml, while 5-HIAA level was unavailable. She had repeated colonoscopy after 2 years with normal neo-ileum, and an octreotide scan 3 years later with no evidence of metastasis.

**Case 6 .**

A 64-year-old male underwent average risk screening colonoscopy. A 10 mm lesion was found on terminal ileal intubation with histopathology revealing carcinoid tumor. His initial lab workup including 5-HIAA and chromogranin was unavailable. A CT scan chest\abdomen\pelvis was performed and showed no evidence of metastasis. The patient underwent a laparoscopic right hemicolectomy, and histopathology confirmed the diagnosis of well-differentiated NET invading the submucosa with metastasis to 2 out of 13 resected lymph nodes (T1N1M0). The patient has not presented for follow-up labs or colonoscopy to date.

**Case 7 .**

A 60-year-old female underwent average risk screening colonoscopy. A 17 mm lesion was found on terminal ileal intubation with histopathology revealing carcinoid tumor. Her initial lab workup revealed a 5-HIAA of 2 mg/gCR and chromogranin A of 559 ng/ml. A CT scan chest\abdomen\pelvis was performed and showed no evidence of metastasis. She underwent robotic ileocolectomy, and histopathology confirmed the diagnosis of insular carcinoid tumor with no evidence of atypia (T2N0M0). A follow-up lab workup revealed a 5-HIAA and chromogranin A of 4 mg/gCR and 149 ng/ml, respectively. She had repeated colonoscopy after 1 year with normal neo-ileum.

**Case 8 .**

A 58-year-old female underwent average risk screening colonoscopy. A 6 mm lesion was found on terminal ileal intubation with histopathology revealing carcinoid tumor. Her initial lab workup regarding 5-HIAA was unavailable and chromogranin A was 48 ng/ml. A CT scan abdomen\pelvis was performed and showed a 1.1 cm soft tissue lesion in the terminal ileum with no evidence of metastasis. A NM octreotide (tumor localization) scan also showed no evidence of metastasis. She underwent an open right-sided hemicolectomy, and histopathology confirmed the diagnosis of well-differentiated carcinoid tumor infiltrating through the subserosal adipose tissue (T3N0M0). A follow-up lab workup revealed a chromogranin A of 56 ng/ml, while 5-HIAA level was unavailable. She had repeated colonoscopy after 1 year with normal neo-ileum

**Case 9 .**

A 69-year-old male underwent surveillance colonoscopy for CRC due to personal history of piecemeal polypectomy. A 15 mm polyp was found on terminal ileal intubation with histopathology revealing carcinoid tumor. His initial lab workup revealed a 5-HIAA of 6 mg/gCR and chromogranin A of 531 ng/ml. A chest CT scan and CT enterography were performed and showed small bowel tumor in the right lower quadrant with peritoneal and omental metastases. He underwent a laparoscopic right hemicolectomy, and histopathology confirmed the diagnosis of transmurally invasive and well-differentiated carcinoid tumor with angiolymphatic and perineural invasion. The tumor revealed metastasis to the mesentery, omentum, and 2 out of 12 resected regional lymph nodes (T4N1M1). The patient has not presented for follow-up laboratory and colonoscopy to date as it has been less than 1 year since initial colonoscopy.

See Table 1 for summary of cases, and Table 2 for colonoscopy and histopathology images.

## 3. Discussion

Gastrointestinal neuroendocrine tumors (GINETs) (also known as “carcinoid tumors”) arise from the enterochromaffin cells and are most commonly located in the small bowel, particularly the terminal ileum. NETs are the most common type of small bowel tumors [1]. Overall, NETs are considered rare, with reported incidence of 6.98 per 100,000. The incidence in the United States and elsewhere has increased significantly due to the increased detection on radiographic imaging and endoscopy. The disease tends to have a slight female predilection and to be more common in the African American population [2, 3].

Patients with small bowel NETs may present with abdominal pain, diarrhea, nausea, and vomiting especially when they have carcinoid syndrome [4]. However, the disease is mostly asymptomatic and patients are usually diagnosed when NETs are incidentally found during routine colonoscopy [5]. The mainstays of testing for small intestinal NETs are urine or serum 5-hydroxyindoleacetic acid (5-HIAA) and chromogranin A (CgA). Urinary 5-HIAA is specific but not very sensitive, as one study reported 100% and 73%, respectively. It is the most sensitive test to diagnose carcinoid syndrome [6, 7]. Serum CgA is a more sensitive but less specific test, as it can be elevated in a wide range of diseases. The level of plasma CgA may correlate with treatment response and have a prognostic value [8]. Its role remains debatable. In the present series, four of the patients had CgA abnormally elevated and urine 5-HIAA was measured in five of the patients with normal initial values. When clinically suspected, imaging is important for localization of the tumor. CT scan, MRI, and diagnostic imaging using radiolabeled somatostatin analogs or gallium (68-Ga DOTATATE) are the primary imaging modalities used.

Although the ileum is the most common site for GINETs, terminal ileal (TI) intubation is not always performed during routine colonoscopy unless ileal pathology such as inflammatory bowel disease (IBD) is suspected. This could be due to time limitations, lack of skills, and presumed unnecessity. On another note, an increase in incidence of rectal NETs has been noticed since the implementation of screening colonoscopy per review of SEER database [9]. The rates at which TI intubation are attempted and completed vary, ranging from 30.9% to 85% [10–13]. The rates among our endoscopists (32% to 82%) are consistent with these reported results.

Patients with locoregional disease are treated with resection of the involved segment and small bowel mesentery even in asymptomatic patients [4, 14]. Technique of resection and adequacy of margins vary according to exact site of disease [15]. The risk of metastasis depends on tumor size. If the tumor is ≤ 1 cm, 1.1–1.9 cm, or ≥2 cm, there is a 12%, 70%, and 85% risk of lymph node involvement, and a 5%, 19%, and 47% risk of distant metastasis, respectively [16]. Our findings somewhat correlate with these numbers. Three patients in our series had tumor size ≤ 1 cm (Case 5, 6, and 8 : 6, 10 and 10 mm, respectively). Two out of three (66.6%) had lymph node involvement, while none (0%) had distant metastasis. Four patients had tumor size 1.1–1.9 mm (Case 2, 3, 7 and 9 : 11, 15, 17, and 15 mm, respectively). Three out of four (75%) had lymph node involvement, and one out of four (25%) had distant metastasis. Two patients had tumor size ≥2 cm (Cases 1 and 4 : 20 mm each). Both (100%) had lymph node involvement, and none had distant metastasis.

In advanced disease, the role of surgery is less clear and reserved for patients without carcinoid syndrome and those who are deemed good candidates for surgical resection. Chemoembolization and systemic therapy are options if surgery is not appropriate. Ten-year survival for patients with small bowel NETs depends on the stage of the disease upon diagnosis. It drops from 95% in stage I to around 40% in stage IV [17]. The significant drop in ten-year survival rates emphasizes the importance of routine TI intubation during routine colonoscopy as it can improve overall outcome. Two of our patients had stage II (22%), six had stage III (66%), and one patient had stage IV (11%). All nine cases had surgical resection of the tumor with eight out of nine having no distant metastasis at the time of resection. Interestingly, the case reported with distant metastasis (Case 9) had initial colonoscopy done 1 year prior to tumor detection where TI intubation had not been performed. A follow-up colonoscopy in 1–3 years was completed in all of our patients who had not had metastasis except one, and all colonoscopies revealed normal neo-ileum.

Our case series is consistent with previous reports by Yarze et al. [18] and Ten Cate et al. [19] and it adds to the sparse literature about TI intubation and highlights the importance of this technique in early detection of small bowel NETs which could potentially affect the outcome. In addition, it is of great importance to mention that the peak age of small bowel NETs is 67–68 years, which is the age by which most patients should have had up to two screening colonoscopies completed [20].

## 4. Conclusion

In conclusion, our case series supports previously published data on terminal ileal NETs incidentally found on screening colonoscopy. Given the minimal increase in procedural time and risk as well as high success rate, the addition of TI intubation to standard screening colonoscopy can be of great added value in the detection of small bowel NETs.

## Figures and Tables

**Table 1:** Summary of all cases.

Patients	Age	Gender	Indication	Size of the lesion (mm)	Urine 5-HIAA (0–14 mg\gCR)		Chromogranin (0–95 ng\ml)		Imaging	Surgery	Pathology	Follow-up notes
					Initial	Follow up	Initial	Follow up				
Case 1	66	Male	Surveillance: personal history of colon polyp.	20	3 mg/gCR	3 mg/gCR	7 ng/ml	40 ng/ml	CT scan: mass in the TI. No findings to suggest metastatic disease	Laparoscopic, switched to mini-laparotomy right hemicolectomy	Low-grade (NET G1) T2N1M0 stage III	Colonoscopy in 2 years: normal neo-ileum
Case 2	57	Female	Average risk screening colonoscopy	11	4 mg/gCR	2.4 mg/gCR	101 ng/ml	99 ng/ml	CT scan: mesenteric nodule measuring 9 mm adjacent to the TI	Laparoscopic right hemicolectomy	Low-grade (NET G1) T2N1M0 stage III	Colonoscopy in 1 and 3 years: normal neo-ileum
Case 3	57	Male	Average risk screening colonoscopy	15	NA	2 mg/gCR	NA	62 ng/ml	CT scan: faint enhancement within the TI. No findings to suggest metastatic disease	Laparoscopic ileocolectomy	Low-grade (NET G1) T4N1M0 stage III	Colonoscopy in 1 and 4 years: normal neo-ileum
Case 4	69	Female	Average risk screening colonoscopy	20	NA	3 mg/gCR	48	70 ng/ml	CT scan: 10 mm lobulated excrescence involving TI. No findings to suggest metastatic disease	Laparoscopic right colectomy with ileocolic anastomosis	Low-grade (NET G1) T1N1M0 stage III	Colonoscopy in 2 years: normal neo-ileum
Case 5	55	Female	Surveillance: personal history of colon polyp	10	3 mg/gCR	NA	1152 ng/ml	1160 ng/ml	CT scan and NM tumor localization: no findings to suggest metastatic disease	Laparoscopic right hemicolectomy	Low-grade (NET G1) T1N1M0 stage III	Colonoscopy in 1 year: normal neo-ileum.
Case 6	64	Male	Average risk screening colonoscopy	10	NA	NA	NA	NA	CT scan: no findings to suggest metastatic disease	Laparoscopic right hemicolectomy	Low-grade (NET G1) T1N1M0 stage III	No follow-up colonoscopy
Case 7	60	Female	Average risk screening colonoscopy	17	2 mg/gCR	4 mg/gCR	559 ng/ml	149 ng/ml	CT scan: no findings to suggest metastatic disease	Robotic-assisted ileocolectomy	Low-grade (NET G1) T2N0M0 stage II	Colonoscopy in 1 year: normal neo-ileum
Case 8	58	Female	Average risk screening colonoscopy	6	NA	NA	48 ng/ml	56 ng/ml	CT scan and NM tumor localization: a 1.1 cm lesion within the TI. No findings to suggest metastatic disease	Open right hemicolectomy	Low-grade (NET G1) T3N0M0 stage II	Colonoscopy in 1 year: normal neo-ileum
Case 9	69	Male	Surveillance: due to piecemeal polypectomy	15	6 mg/gCR	NA	531 ng\ml	NA	CT chest and enterography: RLQ tumor with peritoneal and omental metastasis	Laparoscopic-assisted right hemicolectomy	Low-grade (NET G1) T4N1M1 stage IV	No follow-up colonoscopy

**Table 2:** Colonoscopy and histopathology images.

Patient	Endoscopic image	Histopathology		
Case 1	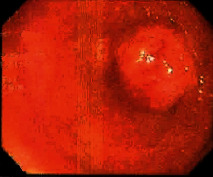 20 mm terminal ileal lesion	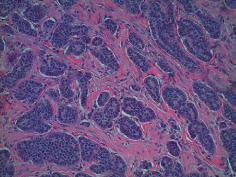 H&E 100x well-differentiated NET	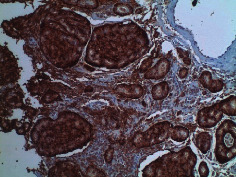 100x chromogranin positive	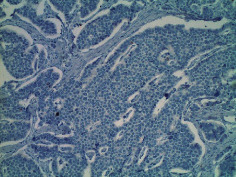 100x Ki-67 proliferation index <2%
Case 2	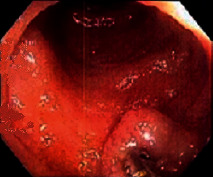 11 mm terminal ileal lesion	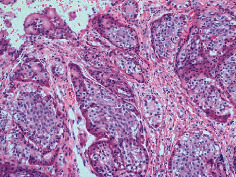 H&E 100x well-differentiated NET	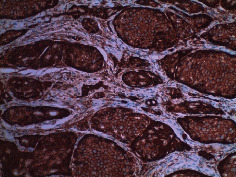 100x chromogranin positive	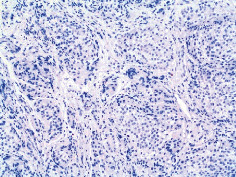 100x Ki-67 proliferation index <2%
Case 3	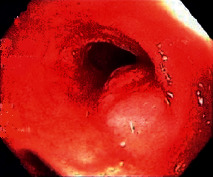 15 mm terminal ileal lesion	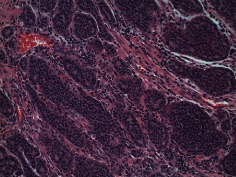 H&E 100x transmurally invasive NET	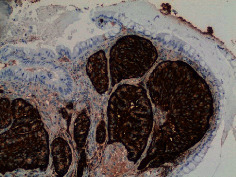 100x chromogranin positive	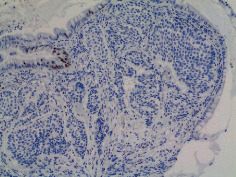 2 100x Ki-67 proliferation index <2%
Case 4	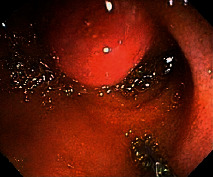 20 mm terminal ileal lesion	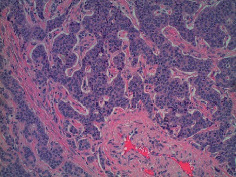 4 H&E 100x well-differentiated NET	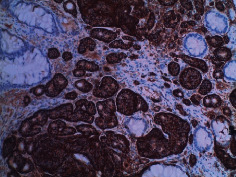 5 100x chromogranin positive	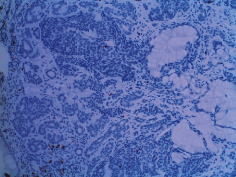 6 100x Ki-67 proliferation index <2%
Case 5	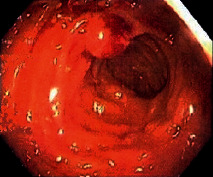 10 mm terminal ileal lesion	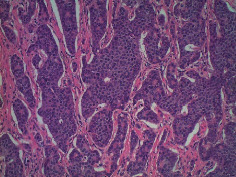 H&E 100x low-grade NET 100x	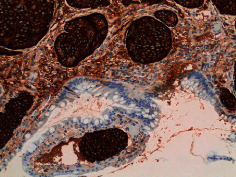 9 Chromogranin positive	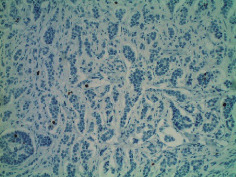 100x Ki-67 proliferation index <2%
Case 6	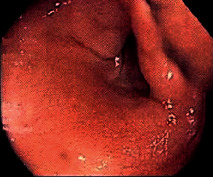 10 mm terminal ileal lesion	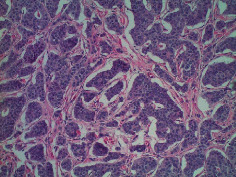 H&E 100x well-differentiated NET	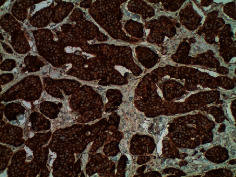 3 100x chromogranin positive 100x	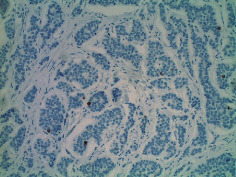 4 Ki-67 proliferation index <2%
Case 7	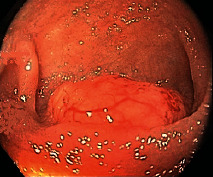 17 mm terminal ileal lesion	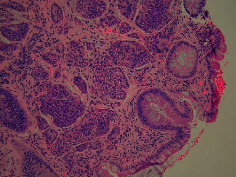 H&E 100x Insular-type NET	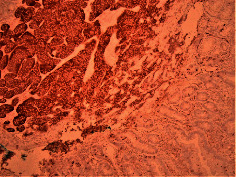 7 No atypia 100x chromogranin positive	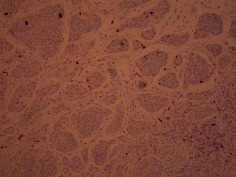 8 100x Ki-67 proliferation index <2%
Case 8	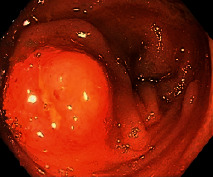 6 mm terminal ileal lesion	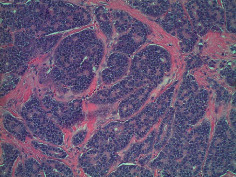 H&E 100x well-differentiated NET	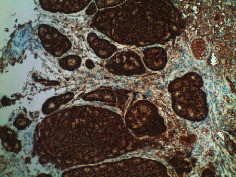 1 100x chromogranin positive	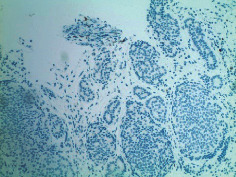 2 100x Ki-67 proliferation index <2%
Case 9	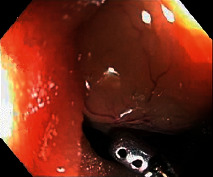 15 mm terminal ileal lesion	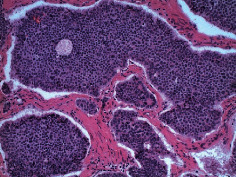 H&E 100x Transmurally invasive NET	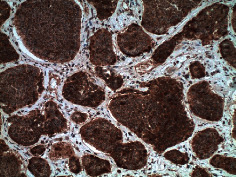 5 100x chromogranin positive	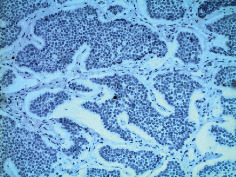 6 100x Ki-67 proliferation index <2%
